# Development of a flexible liver phantom for hepatocellular carcinoma treatment planning: a useful tool for training & education

**DOI:** 10.1186/s41205-024-00228-9

**Published:** 2024-07-22

**Authors:** Abdulla Al-Thani, Abdulrahman Sharif, Sami El Borgi, Shameel Abdulla, Mahmoud Raja Ahmed Saleh, Reem Al-Khal, Carlos Velasquez, Omar Aboumarzouk, Sarada Prasad Dakua

**Affiliations:** 1 Department of Mechanical Engineering, Texas A&M University at Qatar, Doha, 23874 Qatar; 2 Qatar Center for Organ Transplantation, Hamad Medical Corporation, Doha, 3050 Qatar; 3 Department of Surgery, Hamad Medical Corporation, Doha, 3050 Qatar; 4 College of Health and Medical Sciences, Qatar University, Doha, 2713 Qatar

**Keywords:** Liver cancer, Phantom liver, 3D printing, Silicone rubber, Ballistic gel

## Abstract

**Purpose:**

Hepatocellular carcinoma (HCC) is one of the most common types of liver cancer that could potentially be surrounded by healthy arteries or veins that a surgeon would have to avoid during treatment. A realistic 3D liver model is an unmet need for HCC preoperative planning.

**Methods:**

This paper presents a method to create a soft phantom model of the human liver with the help of a 3D-printed mold, silicone, ballistic gel, and a blender.

**Results:**

For silicone, the elastic modulus of seven different ratios of base silicone and silicone hardener are tested; while for ballistic gel, a model using 20% gelatin and 10% gelatin is created for the tumor and the rest of the liver, respectively. It is found that the silicone modulus of elasticity matches with the real liver modulus of elasticity. It is also found that the 10% gelatin part of the ballistic gel model is an excellent emulation of a healthy human liver.

**Conclusion:**

The 3D flexible liver phantom made from a 10% gelatin-to-water mixture demonstrates decent fidelity to real liver tissue in terms of texture and elasticity. It holds significant potential for improving medical training, preoperative planning, and surgical research. We believe that continued development and validation of such models could further enhance their utility and impact in the field of hepatobiliary treatment planning and education.

## Introduction

**Fig. 1:**
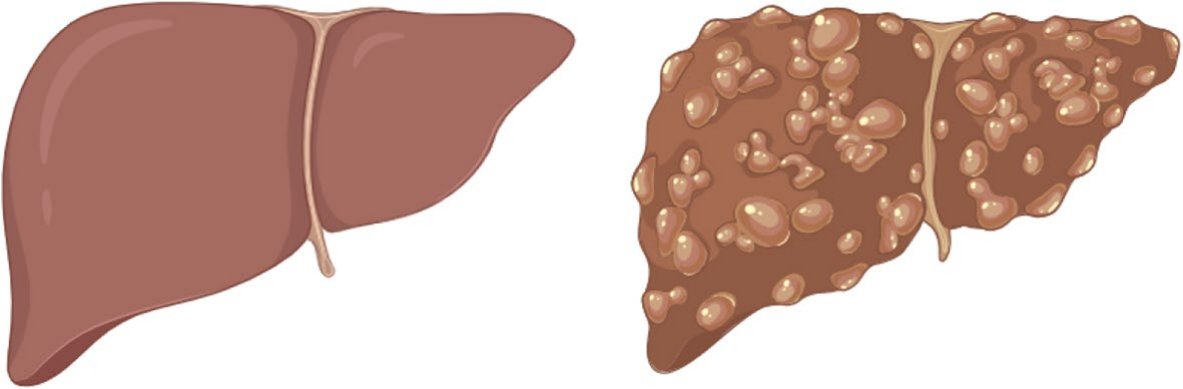
A healthy liver and a liver affected by cirrhosis (created by Biorender https://app.biorender.com)

Liver cancer is one of the most prominent diseases that affect the lives of humans [1–4]; in 2020 alone, liver cancer was the root of nearly 830,000 deaths, projecting it as the third highest cause for mortality, followed by colorectum cancer and lung cancer [5, 6]. Liver cancer seems to become more dangerous as it is expected that the number of deaths will increase by 56.4% by 2040 (around 1.4 million people) if no efforts are planned to address [7]. In Qatar, liver cancer was reported to be the highest mortality rate among the other GCC member countries (which are Saudi Arabia, Kuwait, Qatar, Oman, Bahrain, and the United Arab Emirates) in 2019 [8–10]. Hepatocellular carcinoma (HCC) is one of the most common types of liver cancer, accounting for about 90% of liver cancer cases [11]. Cirrhosis (shown in Fig. 1) has high mortality like HCC [12], moreover, cirrhosis alone is the eleventh most common cause of death, which is a big concern since people with obesity are more likely to be affected by this, and obesity has continuously been increasing [13, 14]. Furthermore, the chronic infections with hepatitis virus and toxicity are the major causes of cirrhosis globally [15, 16].

Cirrhosis increases the chance of developing HCC and is caused by liver damage (such as consuming alcohol and obesity) [12, 13]. There are different treatments depending on the stage of the liver cancer; for the earlier stages, chemotherapy and systematic therapy are usually considered [17]; for mild cirrhosis, surgical resection is an effective treatment [17], whereas for the higher stages, liver transplantation is considered as the most effective.

**Fig. 2:**
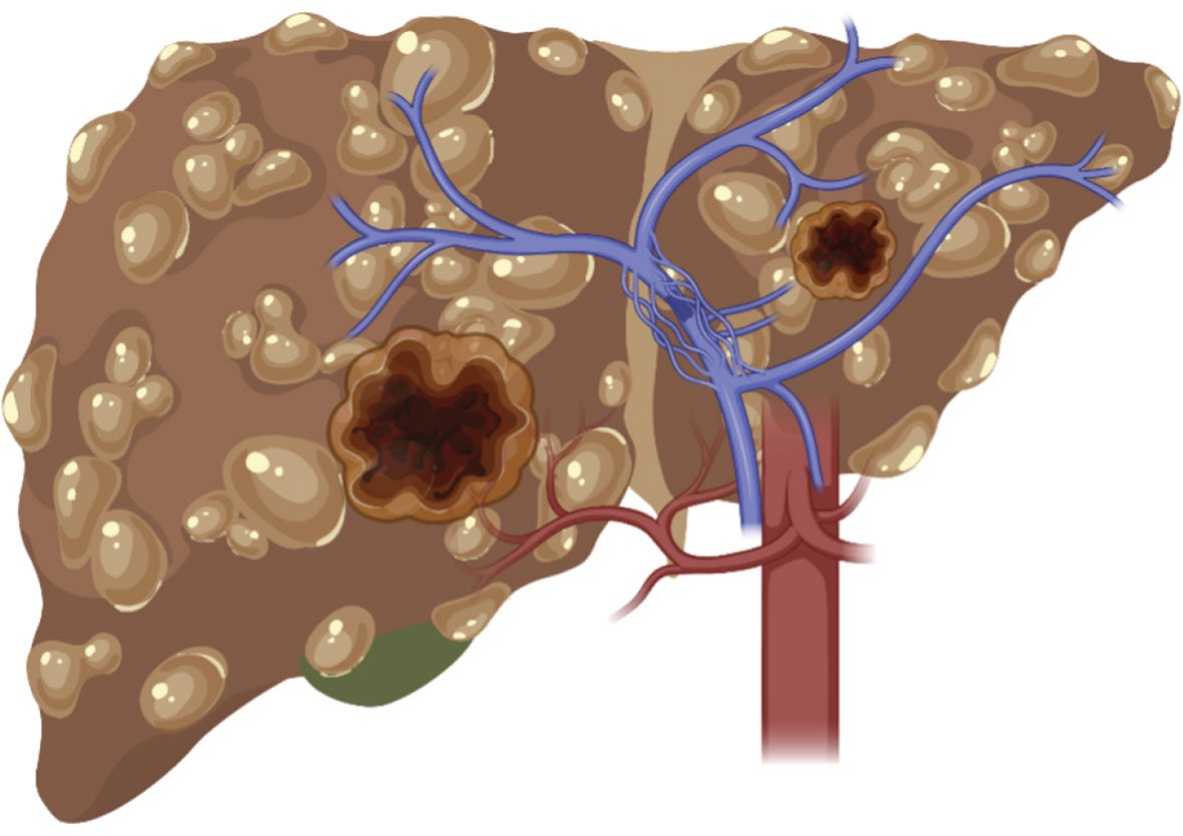
Illustration of a liver with tumors (created by Biorender https://app.biorender.com)

As can be seen in Fig. 2, the tumor(s) are not always easy to be dealt by the clinician to treat. Sometimes, they are in the vicinity of the hepatic artery (colored red) and the portal vein (colored blue); sometimes, they are surrounded by the hepatic vessels and so on. This causes and increases complications for the clinicians, as there is a risk of damaging the nearby arteries and veins. Sometimes, the clinicians want to assess the blood loss due to a particular treatment. The clinicians usually prefer the patient specific physical liver phantom, because the 3D print provides a tangible, detailed replica of the patient’s liver; the interventional radiologits/surgeons can examine the complex anatomical relationships from different angles, which is particularly useful for intricate hepatobiliary treatments. The liver phantom flexibility is another feature that could provide a sense of force feedback to manipulate the scissors’ movement in case of resection [18]. This paper aims to 3D print a liver phantom like real patient using the materials mimicking similar mechanical properties to an actual liver.

Anwari et al. mention that structural, mechanical, and radiological properties of 3D printed models are the main considerations for the selection of materials used in 3D printing of anatomical models, with structural and mechanical properties being especially important for surgical planning [19]. This indicates that the material must have the same mechanical properties as a real liver and so is the technique of creating the mold with similar material ensuring the structure of the liver model same as a real human liver. The modulus of elasticity (E) of a healthy liver is nearly 12.16 ± 1.20 (mean ± SD) and 196.54 ± 13.15 in kPa under axial loading for the tensile and compressive elastic modulus, respectively [20, 21]. For the case of a liver (with a tumor), the tensile elastic modulus is expected to be between 18.25 to 75 kPa, which is at least 1.5$\times$ and, at most, around 6$\times$ the tensile elastic modulus of a healthy liver [22]. This indicates the need for liver phantom material to be flexible and have similar modulus of elasticity. Based on the literature, we prefer silicone initially; however, we have also considered ballistic gel since it is close to that of an actual human organ [23]. The elastic modulus (E) of silicone rubber is nearly 50 MPa [24], with a minimum value of 1 MPa and a maximum of 50 MPa [25]. The elastic modulus of silicone rubber ranges between 0.52 and 62.1 MPa [26]. Because of the extensive range of values in the elastic modulus, the different ratios between silicone rubber (which is referred to as silicone A) and silicone hardener (which is referred to as silicone B) are experimentally calculated, as the extensive range of values could be due to different silicone ratios being used. A ballistic gel is usually used in forensic sciences to test ammunition due to its similarity to that of human skin and organs [23], a potentially suitable material for the liver phantom. The Federal Bureau of Investigation (FBI) recommends using 10% gelatin to 90% water to make ballistic gel; however, the North Atlantic Treaty Organization (NATO) also mentions that the gelatin could be used for ammunition testing up to 20% [27]. There is a clear similarity between the bullet impacting the ballistic gel and the way it vibrates after tearing through when using ammunition on the ballistic gel [28]. This has motivated us to try creating a liver model out of ballistic gel.

Polysiloxanes (Silicone) is a type of polymer with Si-O repeat units [29]. On their own, the covalent bond between Si and O within the chain is strong, however, there exists weak Van der Waals forces between each chain of the silicone polymer [29]. To rectify this, silicone’s chemical structure (and polymers in general) is modified so that cross-linking between chains occurs [30]. The silicone rubber used in this study is a two-part room temperature vulcanizing silicone rubber (RTV-2) cured with platinum as a catalyst for an addition reaction [31]. The part with the platinum catalyst is referred to as Silicone A, and the part with the Si-H crosslinker is referred to as Silicone B in this paper.

## Methodology

### Data description and 3D geometric model

**Fig. 3:**
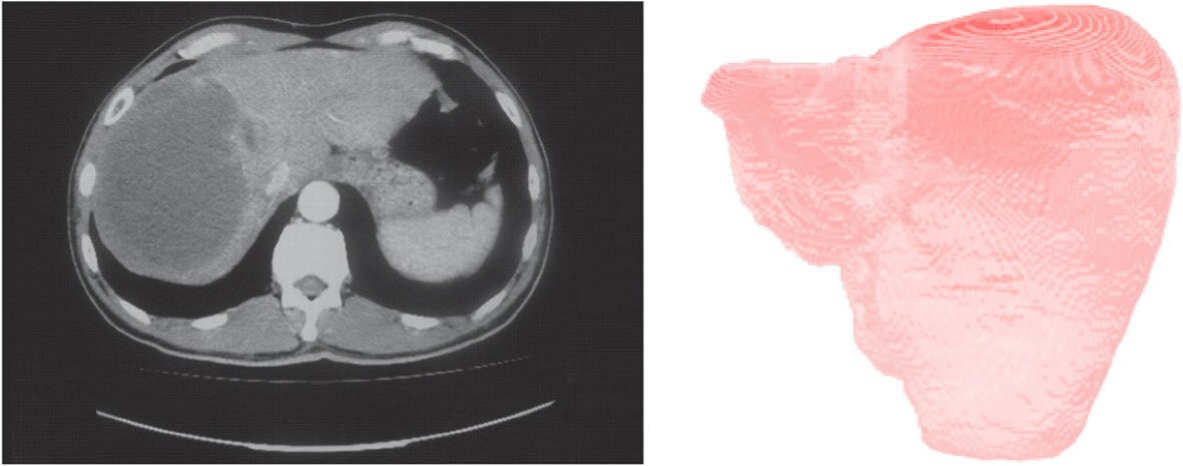
A slice from the input CT volume (left) and the 3D liver model (right)

We have obtained computerized tomography (CT) data from patients treated at Hamad Medical Corporation suffering from HCC. A slice of an input CT volume from a patient is provided in Fig. 3). The spatial dimension of the CT scans is 512$\times$512, with the number of slices in the range of (50 to 1100).

The literature is rich for developing geometric models that include conventional [32–38] and artificial intelligence methods [39–42]. In this work, we have designed a 3D geometric model from the input CT images using a lightweight neural network [43] as shown in Fig. 3. This 3D computer model is used to produce a mold by additive manufacturing, which allows us to obtain flexible phantoms by pouring silicone or ballistic gel materials into the cast mold.

### 3D printing the mold

**Fig. 4:**
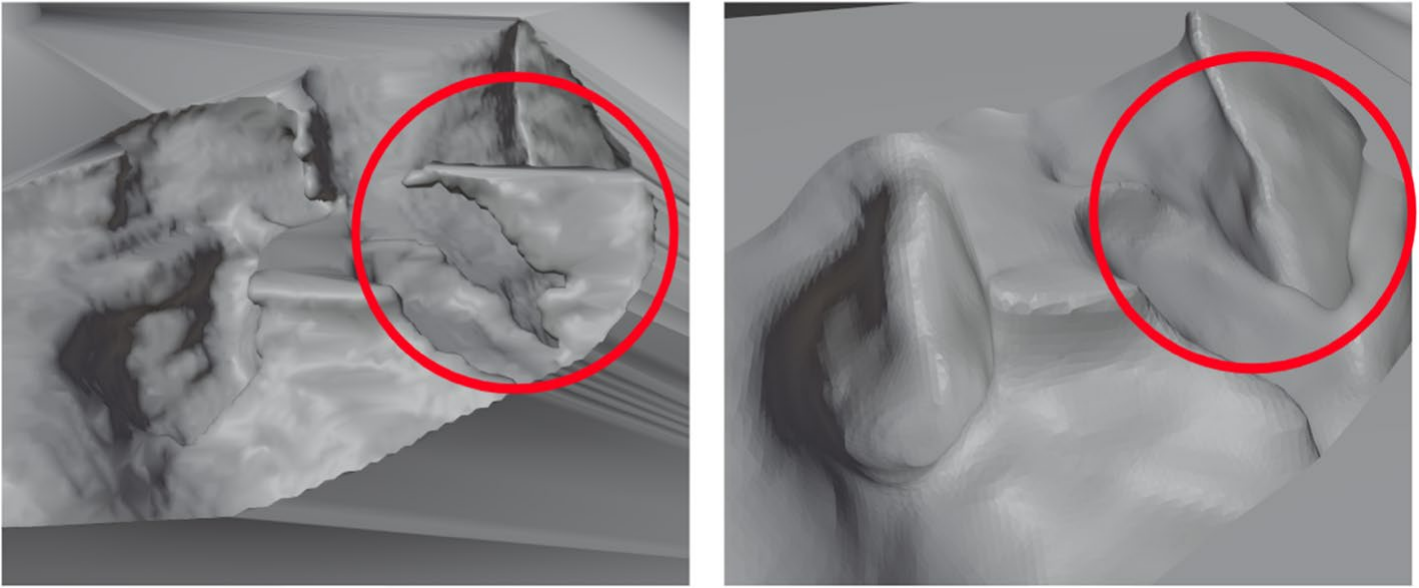
Liver design modifications; (**a**) before smoothing and (**b**) after smoothing

The geometric model had a lot of holes/cavities in the design, which resulted in some problematic overhangs when designing the mold. These overhangs make it harder to 3D print and increase the risk of failure during printing. The mold of the model was generated by subtracting the model from a box volume that served as the mold base. The 3D liver model was imported in a Computer-Aided Design (CAD) and was positioned within the box’s boundaries, ensuring proper alignment. First, SolidWorks was used to adjust the digital liver model [44]; after that, Blender was used to create the mold of the adjusted digital model [45]. Using Boolean subtraction functions of the software, a cavity representing the mold was created by subtracting the model’s volume from the box. Due to the liver model’s complex design, there were many overhangs when the Boolean function was used. These overhangs resulted from the holes/cavities in the given liver model, as shown in Fig. 4. The solution to this problem was to simplify and smoothen the liver design, removing the cavities and making it easier to design the mold and 3D print, as shown in Fig. 4. Although this decreases the accuracy compared to an actual liver, the change appears insignificant and carries a minor effect on the final product.

**Fig. 5:**
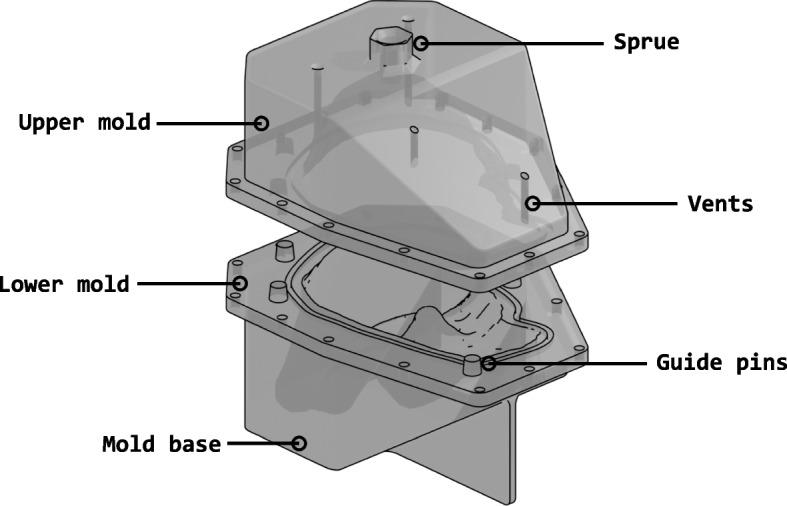
Final design of the two part mold

The initial mold design featured a simple bounding box as the exterior for the liver model. Considering the size of the mold, this results in a considerable amount of material waste during printing. The mold was redesigned to remove the excess material as shown in Fig. 5. This resulted in significant material savings and printing time. The mold design was then fine-tuned by incorporating features like vents and sprue. The vents are small channels or passages placed in the upper mold to ensure air is not trapped in the mold as material is poured into it. The sprue is used to ease the pouring of the different mixtures into the mold without spillage or waste. A few extra modifications were added to ensure a better lineup when closing the mold, like the guide pins, which guide the top half of the mold to connect perfectly to the bottom half. In addition to the guide pins, extra holes on the outside were added to tightly seal the two halves together. The liver mold was ready to be 3D printed using Acrylonitrile Butadiene Styrene (ABS) plastic.

### Creating the silicone model

With the 3D-printed mold was ready, the next step was to use a silicone rubber mixture consisting of solutions A (the base silicone material) and B (the hardener). We followed a trial-and-error approach to find an optimal ratio of A and B as needed to match the liver’s consistency. The safety data sheets were printed for silicone rubber [46], silicone spray [47], and thinner [48] to ensure safety before the printing process. The densities of A and B were assumed to be the same and were verified to be the same by measuring the total mass of the container containing silicone A and silicone B. Solution B initiates a chemical reaction when combined with solution A, which causes the base silicone material to harden. The properties of the resulting silicone vary depending on the ratios of the two mixtures. Before proceeding with the silicone mixing, the dimensions of the ice tray that stores each mixture were measured by a caliper to find a length of 49 mm, width of 28 mm, and depth of 26 mm for all the testing samples.

**Table 1:** Mass needed to fulfill each ratio of A:B that was tested

A : B	10 : 1	5 : 1	2 : 1	1 : 1	1 : 2	1 : 5	1 : 10
Solution A (in grams)	90.9	83.3	66.7	50	33.3	16.7	9.1
Solution B (in grams)	9.1	16.7	33.3	50	66.7	83.3	90.9

Seven ratios of A:B were chosen to be 10:1, 5:1, 2:1, 1:1, 1:2, 1:5, and 1:10. Each mixture was ensured so that both ratio conditions were fulfilled, and the total mass was added up to 100 g. The amount of each solution added (by mass) is shown in Table 1. The mass of each ratio solution was carefully added using a precision scale to ensure the masses were added correctly by using Table 1. Starting with the 10:1 ratio, solution A was poured into a paper cup (in which the precision scale resets the cup’s weight) until it reached 90.9 g, and then 9.1 g of solution B was added until the total mass of solution reached 100 g. The solution mixture was properly mixed for around 5 minutes before pouring into the ice tray. In case the solution fails, each column in the ice tray includes two samples. Similarly, the other ratios followed the same process.

The samples were left to solidify for a period of four days. A compression test was then performed using a PASCO stress-strain apparatus (AP-8214B) [49]. The setup uses a hand crank with a pitch of 1 mm for pushing the sample from one side. A PASCO rotary motion sensor (PS-2120A) with an angular resolution of 0.09^∘^ was used to track the strain in the samples [50]. At the same time, a PASCO force sensor (PS-2104) with a range of $\pm 50$ N and resolution of 0.03 N was used on the other side of the sample to measure the force [51]. The sensors’ output is recorded and displayed on the computer with the software, LoggerPro [52]. A steady rotational movement was maintained to obtain accurate results during this test since failing to do so results in data with noise. The test was done for all 14 samples, resulting in 14 sets of data measurements (two sets of data for each ratio). The data sets were saved as CSV files to be easily read. Since the linear displacement was directly correlated to the rotation, it was converted to mm by dividing the rotation angle by one revolution per mm (360 degrees per mm). The strain was calculated by dividing the displacement by the length of the sample. The stress was calculated by dividing the force’s negative value by the sample’s cross-sectional area since a negative value would indicate that the sample was being compressed. The area is estimated by multiplying the width with the depth of the sample. After finding the stress and strain of the samples, the next step was to plot a stress-strain curve for each sample and estimate the modulus of elasticity of the sample. After that, two ratios that had the closest modulus of elasticity for liver and tumor, as based on the literature review, were selected to fill the mold. Using the CAD design, the volume of the model was found to be 1811 mL, and by splitting the volume to have 20% tumor and 80% liver, an approximate amount of mass needed for each ratio was found. The density of solutions A and B were calculated by measuring 220 g for a solution of 200 mL, which indicated that the density was 1.1 g/mL. The tumor part of the model was added first by recreating the selected ratio sample (mentioned in the “Results” section) with a volume of 360 mL and pouring it slowly into the mold to avoid air bubbles. The mold was placed inside a vacuum chamber for around 5 minutes to remove the air bubbles formed when mixing the ratio. Next, the liver part of the model was created by creating a 1450 mL mixture with the selected ratio, leaving it inside the vacuum for a few minutes before pouring it inside the mold. If the silicone mixture is left outside for a while, its viscosity increases rapidly, which results in difficulties pouring the mixture into the mold. Therefore, both the tumor and liver parts were prepared with haste before either solidifies. After the liver mixture was left in the vacuum chamber for 5 minutes, it was poured inside the mold containing the tumor part. Finally, the liver mold was solidified, and both parts stuck together firmly without losing their properties.

### Creating the ballistic gel model

The experimental setup consists of measuring cups, a precision scale, unflavored gelatin, red food coloring, a kettle to prepare hot water, and petroleum jelly. The liver mold was cleaned to fabricate the gelatine model. The petroleum jelly was then applied on the inside of the mold so that the ballistic gel mixture did not stick to the mold’s surface, allowing the model to be easily removed after it had been prepared. The assumption of the tumor being around 20% of the liver in the silicone section persists through the ballistic gel experiment. By adding 90 g of gelatin, the volume was found to be 110 mL, which indicated that the gelatine had a density of around 1.2 g/mL. Therefore, since the volume of the mold was about 1800 mL, the weight of the mixture was assumed to be about 2000 g when accounting for the water density as 1 g/mL. Then, the tumor part was prepared by adding gelatine (80 g) to the measuring cup placed at the scale and filling the scale with hot water until the scale showed 400 g (which indicated that the conditions of the tumor being 20% of the liver and the ballistic gel concentration being 20% gelatine and 80% water). After adding red food coloring, mixing the ballistic gel mixture, and scooping off the foam, the mixture inside the measuring cup was poured inside the liver mold and was left in the freezer for about 20 minutes. After that, the mold was moved to a refrigerator at 3^∘^C for around 3 hours, allowing the ballistic gel to solidify. After the tumor section solidified, the rest of the liver was prepared by splitting 160 g of gelatine evenly between two measuring cups (80 grams of gelatine between both measuring cups) and then adding hot water to both cups until the weight of the mixture at each measuring cup both reaches 800 g (indicating that both cups have a mixture of 10% gelatine and 90% water). After mixing the measuring cups and scooping off the foam, the mixtures were poured onto the liver mold until the mold was filled. Similarly, the mold was left in the freezer for about 20 minutes and then moved to a refrigerator at 3^∘^C for around 3 hours to allow the entire mixture to solidify.

## Results

This section provides all the intermediate outcomes and results, including the mold and 3D-printed liver. The elastic modulus of each ratio and the stress-strain curve have also been included.

### 3D Printing the mold

**Fig. 6:**
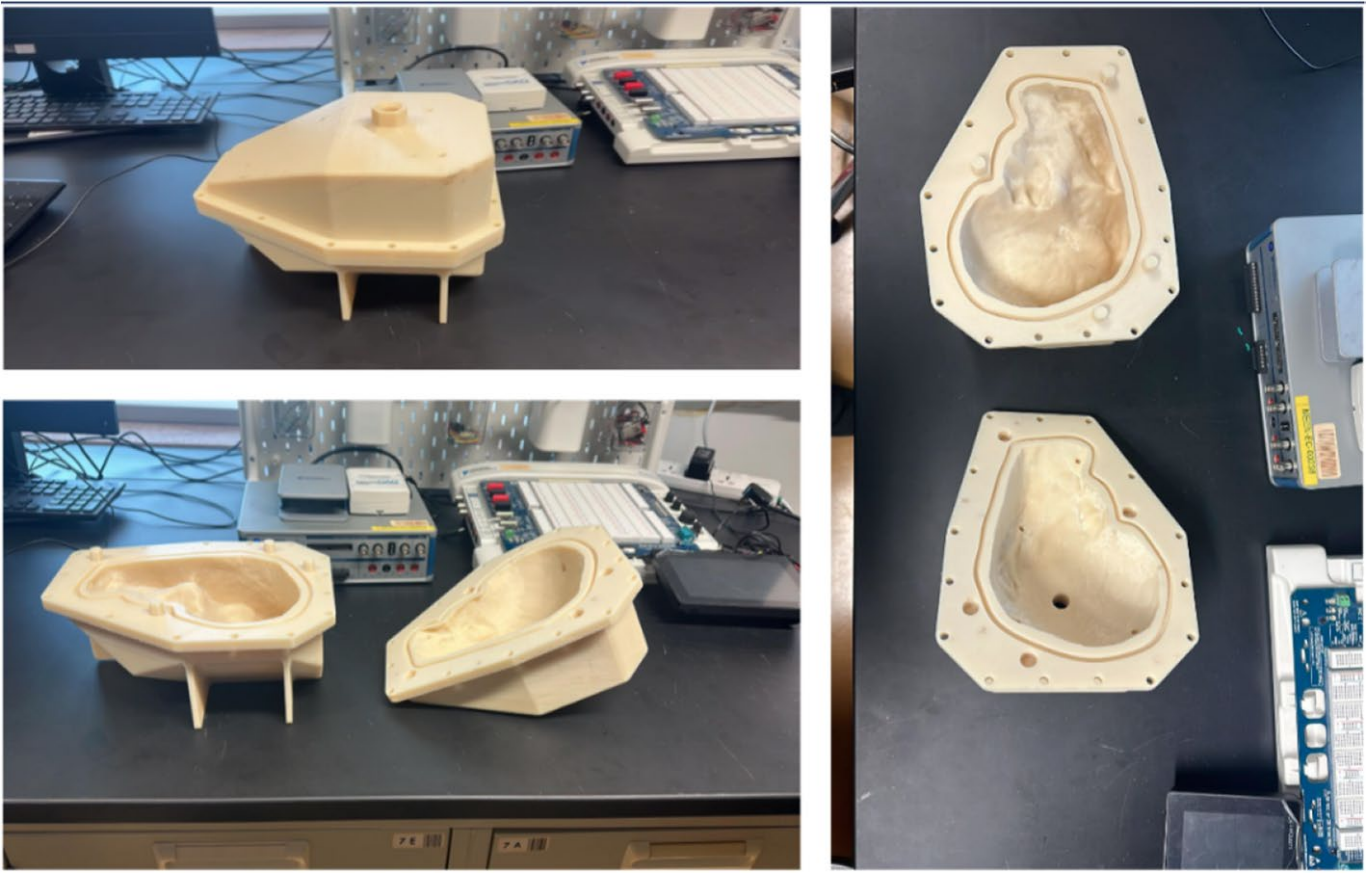
3D printed liver mold

As shown in Fig. 6, the liver mold no longer has any extra unnecessary space. The liver mold has a funnel to pour the mixture into the mold. It has four small vents on the upper half of the mold for air to escape and guiding pins so that the upper half fits with the bottom half of the mold. Since it was printed using ABS plastic, it has excellent water resistance; thus, leaking from the print itself was not an issue.

### Silicone and ballistic-gel model

**Fig. 7:**
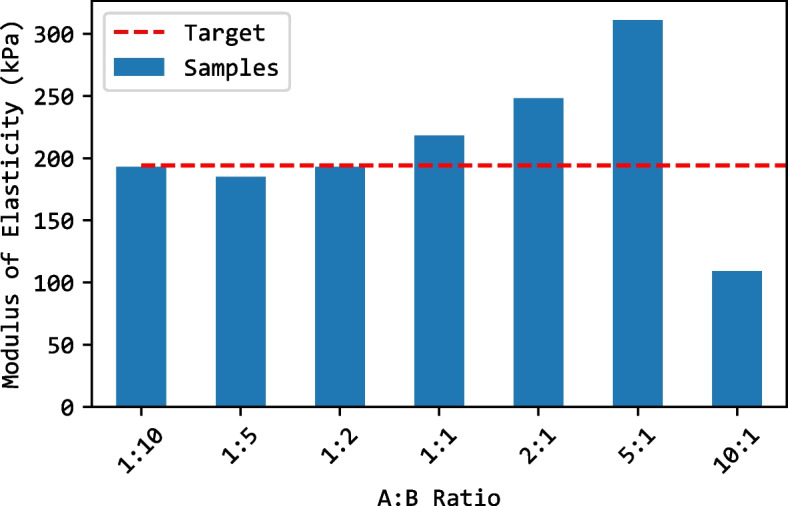
Modulus of elasticity of silicone samples with various A:B ratio

**Fig. 8:**
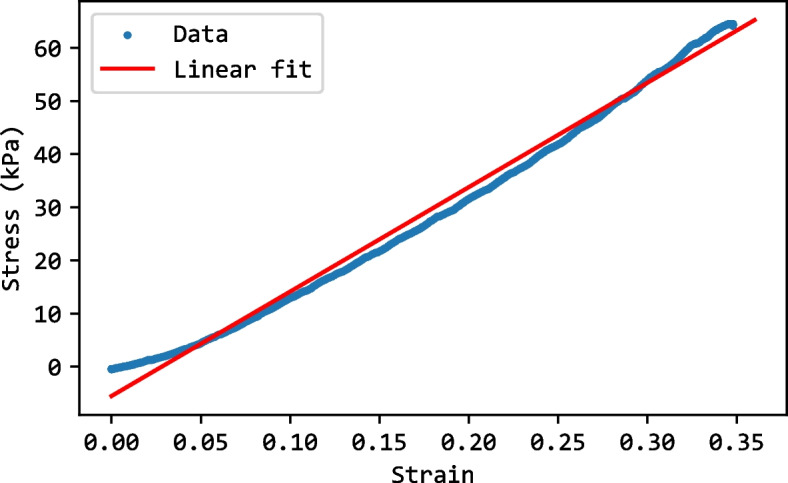
The stress-strain curve of a sample prepared with A:B ratio of 1:2

**Fig. 9:**
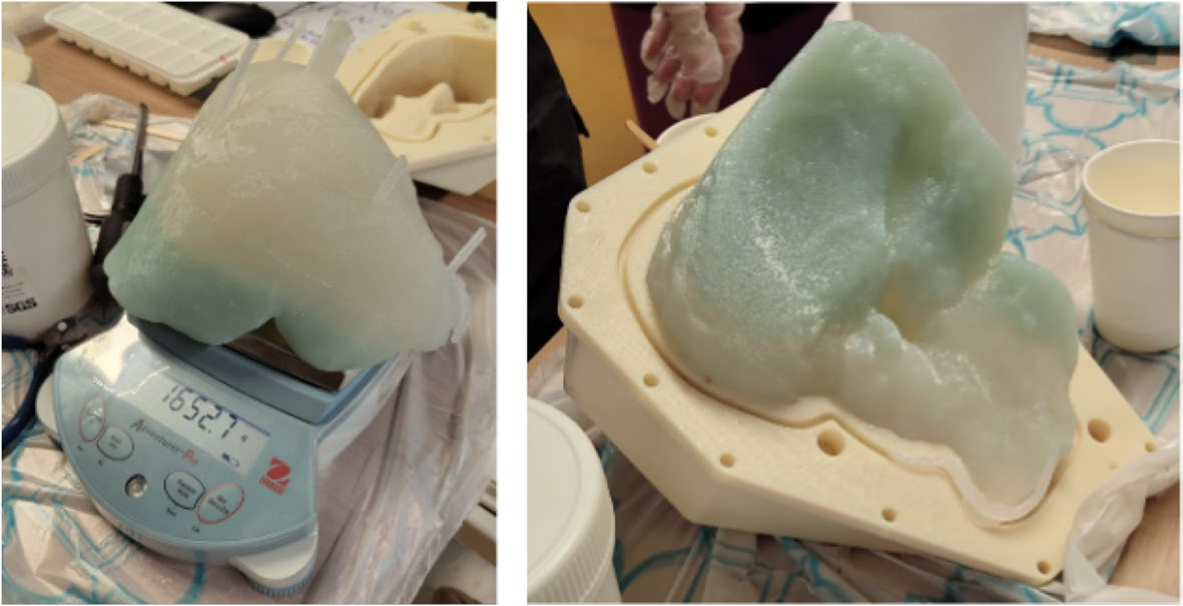
Silicone rubber liver model showing the green color as the tumor

**Fig. 10:**
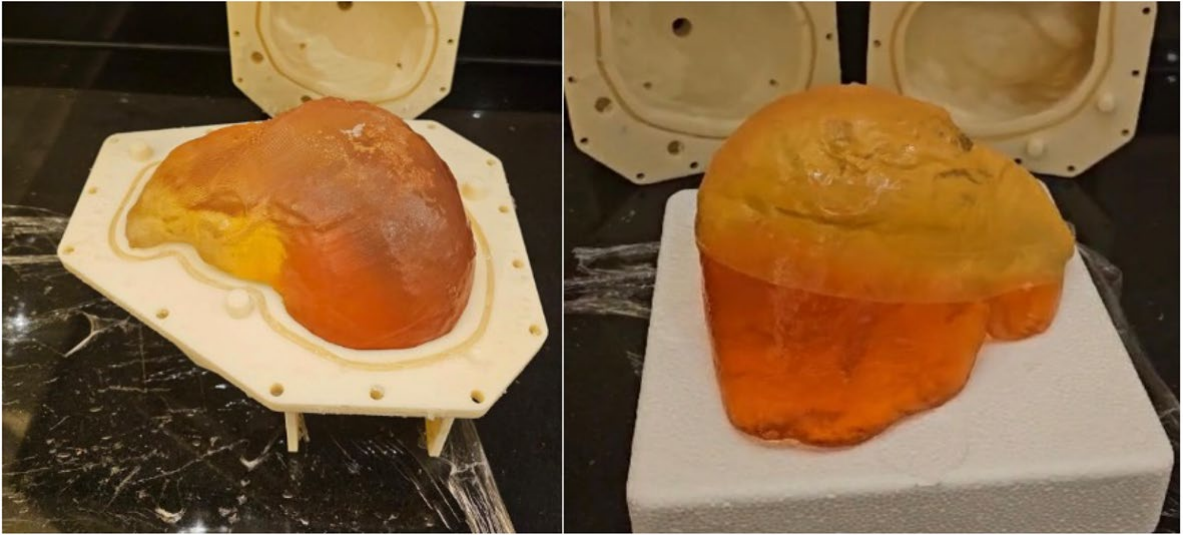
The ballistic gel model in its mold (left) and the full liver model with a visible red tumor (right)

Figure 7 shows that the ratio 5:1 has the highest elastic modulus compared to all the other samples, while 10:1 has the lowest elastic modulus. Considering the value of human liver elastic modulus as 196.54 KPa [20, 21], the ratio of 1:2 was the closest. Therefore, a ratio of 1:2 was chosen for the solution of the healthy liver tissue. Since the tumor is usually harder than the liver, the ratio 5:1 was used due to its higher elastic modulus value. Figure 8 shows the stress-strain plot for a sample with a 1:2 ratio, illustrating how the modulus of elasticity was estimated from the slope. Figure 9 shows the final solidified liver model made from silicone rubber. It shows the separation between the healthy liver tissue(white) and the tumor tissue(green). The total mass of the liver model is 1652.7 g. The extra material from the vents and sprue was removed since they were not included in the model design. Upon evaluation, clinicians at Hamad Medical Corporation (HMC) found the model excessively stiff. This feedback suggests a need for further investigation into the discrepancy between the expected and observed mechanical properties of the silicone used. Meanwhile, we opted to create a model using gelatine; this alternative model is illustrated in Fig. 10.

To establish the clear criteria for what constitutes an “excellent approximation” of liver tissue, we follow the below criteria: (a) Texture and Consistency: How similar is the tactile feel to real liver tissue? (b) Elasticity: Does the material mimic the elasticity of liver tissue when compressed or manipulated? (c) Cutting and Suturing Properties: How well does the material could simulate the experience of cutting, suturing, or other surgical manipulations? Next, we create a Likert scale for each criterion with a range of responses that include: strongly disagree, disagree, neutral, agree, strongly agree. We have drafted a questionnaire that includes statements related to each evaluation criterion and the clinicians have rated their agreement with each statement on the Likert scale. The questions were: 1) “The texture and consistency of the 10% gelatin-to-water mixture closely resemble that of actual liver tissue.” 2) “The elasticity of the 10% gelatin-to-water mixture is comparable to that of real liver tissue.” 3) “The 10% gelatin-to-water mixture simulates cutting and suturing properties of liver tissue effectively.” We have selected a group of three clinicians with varying experience in hepatobiliary surgery (in the range 5-9 years) for this validation, who touched the printed phantom and responded to the questions. According to the clinicians, the 10% gelatine to water (the orangish-yellow region above the red tumor part in Fig. 10) is an excellent approximation of an actual liver. Furthermore, the same protocol has been followed for 20% gelatine to water (the red tumor region in Fig. 10) against the liver tumor that the clinicians have found satisfactory approximation.

## Discussion and future work

### Discussion

When comparing the results between the silicone and ballistic gel models, the latter was found to be more-preferred option. A phantom of ballistic gel matches the liver well and is much cheaper and less complicated to produce. Another advantage of the ballistic gel model over the silicone one is that after the tumor in the organ is surgically removed, the model can be updated by leaving the tumor part in a pot surrounded by hot or boiling water to melt without scorching [28]. 3D-printed models of human organs are currently gaining increased attention and a lot more development is still needed for surgical planning [53–55]. As of now, research regarding 3D-printed liver models tends to be more focused to better visualize the patient’s liver (instead of relying on 2D images from X-ray scans, for example) [19, 56].

#### Limitations and challenges

Poisson’s ratio was not accounted for when calculating the modulus of elasticity of the silicone samples. Silicone rubber is versatile; its extended curing period compared to other materials can potentially slow down production schedules, impacting the timeline. Silicone rubber is a hydrophobic material with an absorption of around 1% [57]. However, if silicone is exposed to high-pressure steam at a temperature above 120^∘^C silicone would deteriorate due to hydrolysis [58]. Since the silicone models are intended for single use for training and planning, storage solutions and sterilization methods have not been considered in this paper. The ballistic gel needs to be stored in a cool and dry place preferably; the calculation of its modulus of elasticity is complex, as standard axial load tests may not be suitable for a material that can easily deform (which means that the Poisson ratio that was ignored before would have an even more significant impact). There were several challenges during the experiment. In terms of 3D printing, the main challenge was to achieve precise dimensions and tight tolerances when designing the 3D model. Failing to do so would have affected the accuracy of molding parts, making the mold’s top and bottom halves unable to connect. For both silicone and ballistic gel, it was hard to introduce the tumor in a way that was not at the bottom or top of the model, meaning that there was not much freedom in creating a complex combination of liver and tumor models without having a straight-line transition. Furthermore, the time was crucial when the silicone mixture became ready to pour. Therefore, little time was spared to vacuum the silicone and remove the bubbles before pouring it into the mold; thus, the silicone mixture became viscous. However, since the silicone mixture is viscous, it was hard to spill from the gaps of the liver mold, unlike ballistic gel, which was able to spill through the gaps in the mold before repeating the process, ensuring the gaps and holes in the mold is sealed with duct tape twice in case it spills through the first layer.

### Future work

The silicone rubber becomes stiffer (not by a large margin) when the temperature exceeds 0^∘^ and reaches about -60^∘^ [59]. This indicates that the modulus of elasticity of silicone rubber can’t probably be modified substantially. Another way could be to reduce the amount of silicone B (hardener) to A, which tests the ratios beyond 10A:1B (such as 20A:1B). For the ballistic gel, the mechanical property cannot be easily found since it is fragile-slumps under its weight, indicating that standard axial tests would not be reliable [60]. However, there are other methods to test ballistic gel, such as rheology tests, first-order Ogden constitutive model, and finite element modeling [60]. It is feasible to apply the control theory to a closed loop with different actuation and sensing attached to a mass-spring-damper mechanical model of the proposed phantom to control its deformation. This process will address the problem of having a real-time deformable phantom liver capable of emulating with precision the effect of the natural breathing process and interaction with other organs. The parameters of the mechanical model can be determined using high-speed cameras and motion trackers to record the displacement of points on its surface. At the same time, finite-element analysis would be helpful to determine the characteristics of the overall forces involved.

## Conclusion

This paper discusses a clinical application of 3D printing strategizing treatments for liver cancer such as hepatocellular carcinoma. When designing the 3D liver mold, we found some necessary additions to ensure a clinically suitable mold design. These additions involve funnels, vents, guiding pins, and holes to tighten the pieces. The design came with some challenges related to surface finish and dimensional accuracy. Two district tests were conducted to assess its efficacy in molding applications, and it was found that the 1:2 ratio is suitable for the liver part of the model. In contrast, the 5:1 ratio is perfect for the tumor part. The second test involved filling the same 3D-printed liver mold with ballistic gel. The 10% gelatine to water ratio, representing the liver region, was considered a close approximation to an actual liver. Similarly, the 20% gelatine to water ratio, depicting the tumor region, was considered a satisfactory emulation of a liver tumor. It is found that the ballistic gel model is sensitive to environmental conditions. When the two tests were compared, the ballistic gel emerged as a more favorable choice for the liver phantom. The ballistic gel model has also been verified by clinicians and proved effective. This is cost-effective requiring only gelatin and water as compared to expensive silicone.

## Data Availability

No datasets were generated or analysed during the current study.
